# Don’t Hide Your Light under a Bushel

**Published:** 1898-04

**Authors:** 


### DON'T HIDE YOUR LIGHT UNDER A BUSHEL.

THE recent action of the Iowa State Association in officially deciding to deny to the journals their transactions, reports, and papers, because the latter cannot furnish extensive reprints without cost, in justice to themselves, and the adoption of a muzzling order upon all those who present papers, reports, etc., and forbidding their copyrighting the same as individuals, or placing in copyrighted publications, is a narrow and very selfish aspect of professional brotherhood and duty. It is a breach of the highest and best professional etiquette, and a denial to their professional brethren beyond the confines of their own State that which every fraternal thought and spirit commands as a duty one to another. This is specially so when the history of every journalistic effort in this country has been one of self-sacrifice on their behalf, without a dollar of return; nay more, the journals of this country have been published at a loss of thousands of dollars to their projectors, not to speak of the time and effort given by editors and promoters without a dollar of compensation, and personal sacrifice of hours of time that would have brought money had they assumed so selfish a spirit or taken so narrow a view of the welfare of the profession and its advancement. Wipe this obnoxious resolution from your records. Veterinary journalism is yours to support and advance. Let your light shine out for the whole world to see and profit by. Sustain every ethical requirement. Your society is not a trades union. While organized specially for Iowans, in general, it is for the whole veterinary world.

